# Hematological Markers as Predictors of ICU Admission in COVID-19 Patients: A Case-Control Study From a Tertiary Hospital

**DOI:** 10.7759/cureus.64213

**Published:** 2024-07-10

**Authors:** Nor Hayati Ismail, Alaa Siddig, Muhammad ‘Akif Hasenan, Majdan Ramli, Noor Haslina Mohd Noor, Mohd Nazri Hassan, Muhammad Farid Johan, Marini Ramli, Rosnah Bahar, Shafini Mohamed Yusoff

**Affiliations:** 1 Department of Hematology, School of Medical Sciences, Universiti Sains Malaysia, Kota Bharu, MYS; 2 Department of Pathology, School of Medical Sciences, Universiti Sains Malaysia, Kota Bharu, MYS; 3 Department of Pathology, Hospital Raja Perempuan Zainab II, Kota Bharu, MYS

**Keywords:** white blood cell, predictor, immature granulocyte, lymphocyte, neutrophil, hematological parameters, survival status, prognostic factors, covid-19, icu admission

## Abstract

Background: COVID-19 illness severity ranges from mild- to life-threatening cases necessitating critical care. Rapid prediction of disease severity and the need for critical care support in COVID-19 patients remain essential, not only for current management but also for preparedness in future pandemics. This study aimed to assess hematological parameters as predictors of intensive care unit (ICU) admission and survival in COVID-19 patients, providing insights applicable to a broad range of infectious diseases.

Methods: A case-control study was conducted at Hospital Raja Perempuan Zainab II, a tertiary referral hospital in Kelantan, Malaysia, from March 2020 to August 2021. Demographics, clinical, and laboratory data were retrieved from patients' medical records. Statistical analyses, including the Chi-square (χ2) test, independent t-tests, and simple and multiple logistic regressions, were used to analyze the data. A receiver operating characteristic (ROC) curve analysis was conducted to assess the accuracy of the predictors.

Results: The median age was 51 years, with females comprising 56.7% (n=148) and males 43.3% (n=113). A total of 88.5% of patients were admitted to non-ICU wards, with a mortality rate of 5.7%. Significant differences were observed in the distribution of hematological parameters between ICU-admitted and non-admitted patients. Neutrophil (OR: 23.96, 95% CI: 7.296-78.675) and white blood cell (WBC) count (OR: 36.677, 95% CI: 2.086-644.889) were the most significant predictors for ICU admission and survival, respectively.

Conclusions: WBC and neutrophil counts exhibited high predictive value for ICU admission, while WBC, neutrophil, lymphocyte, and immature granulocyte (IG) counts were significant predictors of survival status among COVID-19 patients. These findings underscore the continued relevance of hematological markers in managing severe respiratory infections and improving critical care triage, with implications for current and future healthcare challenges.

## Introduction

COVID-19 symptoms vary widely, from asymptomatic cases to life-threatening illness requiring intensive care unit (ICU) admission [1]. Studies in the United States have shown a surge in demand for ICU beds due to the increasing number of COVID-19 cases, raising concerns about adequate patient accommodation. Factors such as age, gender, and comorbidities contribute to disease severity and ICU admission probability [2]. In addition, ICU care has been instrumental in reducing COVID-19 mortality rates [3]. Hematological laboratory data has emerged as a valuable tool for assessing ICU admission risk, reflecting inflammatory response, coagulation abnormalities, and immune dysregulation [4]. Previous studies showed that COVID-19's pathogenesis is associated with hematological abnormalities, including bone marrow cell infection [5] and increased neutrophil counts and lymphopenia are linked to disease severity and mortality [6].

The ability to accurately predict which COVID-19 patients will require ICU admission is critical for optimizing healthcare resources and improving patient outcomes. Recent studies have highlighted the potential of hematological parameters as predictors for disease severity and ICU admission in COVID-19 patients. These parameters, including white blood cell (WBC), immature granulocyte (IG) counts, neutrophil counts, and lymphocyte ratios, offer valuable insights into the progression of the disease [7,8]. However, the exact relationship between these hematological markers and the need for ICU admission and patient survival remains unclear, necessitating further investigation.

Understanding the predictors of ICU admission among COVID-19 patients is essential for several reasons. It allows healthcare providers to identify high-risk patients early, ensuring timely and appropriate interventions. Additionally, it aids in the efficient allocation of critical care resources, which is particularly important during peak infection periods when healthcare systems are overwhelmed.

This study aims to fill the gap in existing research by comparing hematological parameters between COVID-19 patients admitted to the ICU and those who were not. By identifying key predictors of ICU admission and survival status, this research seeks to enhance clinical decision-making and improve patient management strategies during the ongoing pandemic.

## Materials and methods

Patients' recruitment

This is a case-control study conducted at Hospital Raja Peremapuan Zainab II, Kota Bharu, Malaysia from March 2020 to August 2021. A total of 261 COVID-19-infected patients, regardless of gender or age, were included in the study. Patients previously admitted to other institutions due to COVID-19 were excluded. The study received approval from the Human Research Ethics Committee, Universiti Sains Malaysia (protocol number: USM/JEPeM/COVID19-44). Patient consent was waived by the Ethics Committee, due to the use of de-identified health record data.

Diagnosis of COVID-19 was confirmed using reverse transcription-quantitative polymerase chain reaction (RT-qPCR) or Rapid Test Kit (RTK) antigen tests on nasal and/or oropharyngeal swabs. Patients were divided into two groups based on ICU admission status. Demographic information, risk factors, clinical data, and survival status were extracted from medical records. Survival status was defined within a 30-day period, categorized as alive or deceased. All cases were further categorized into five clinical stages: stage 1 (asymptomatic), stage 2 (symptomatic, no pneumonia), stage 3 (symptomatic, pneumonia), stage 4 (symptomatic, pneumonia, requiring supplemental oxygen), and stage 5 (critically ill with multiorgan involvement). Disease severity for ICU admission was assessed based on each patient's clinical status using these clinical stages.

Sample processing

Three milliliters of venous blood were collected in ethylenediaminetetraacetic acid (EDTA) or dipotassium EDTA (K2-EDTA) tubes from each patient. The tubes were gently inverted to ensure thorough mixing with the anticoagulant, preventing clot formation and ensuring homogeneous distribution. The samples were analyzed using a hematological automated analyzer (Sysmex Corporation, Kobe, Japan). Routine hematological parameters such as WBC count, hemoglobin level, platelet count, and differential counts were measured. Additionally, extended hematological parameters including IG count, high fluorescence lymphocyte cell count, and various ratios such as neutrophil-to-lymphocyte ratio (NLR), lymphocyte-to-monocyte ratio (LMR), and platelet-to-lymphocyte ratio (PLR) were assessed in this study.

Data analysis

Statistical analysis was conducted using Statistical Package for the Social Sciences (IBM SPSS Statistics for Windows, IBM Corp., Version 26.0, Armonk, NY) to compare ICU and non-ICU groups. Descriptive statistics summarized baseline characteristics, with mean and standard deviation for continuous variables and frequency tables for categorical variables. Categorical variables were compared using Chi-square or Fisher’s exact test, while continuous variables were analyzed using independent t-tests. Results were presented as a mean with a 95% confidence interval (CI), and p-values <0.05 were considered statistically significant.

Simple and multiple logistic regressions were performed to identify predictors for ICU admission and survival status among COVID-19 patients, focusing on hematological parameters. Multiple logistic regression was used to develop the final predictive model, with variables reaching statistical significance (p < 0.05) including forward and backward stepwise selection. This analysis aimed to determine the association of combined hematological parameters with ICU admission and survival status after 30 days of COVID-19 infection.

The model evaluation utilized receiver operating characteristic (ROC) curves and the calculation of the area under the curve (AUC), along with the determination of the 95% CI. ROC curves assessed the accuracy of key variables in predicting ICU admission and survivability.

## Results

This study included 261 patients, with a median age of 51 years. Females accounted for 56.7% (148/261), while males comprised 43.3% (113/261). Most patients (88.5%, 231/261) were admitted to non-ICU wards, with 11.5% (30/261) admitted to the ICU at stage 4 or stage 5. Among non-ICU patients, most were in stages 1-3, with 37.7% (87/231) at stage 4. Table 1 summarizes the demographic and clinical characteristics of the 261 patients. In the non-ICU group, females predominated (58%, 134/231), while males were more prominent in the ICU group (53.3%, 16/30). The majority of ICU patients (73.3%) were over 55 years old, whereas the non-ICU group showed a more even distribution across age groups, with the lowest rate (2.59%, 6/231) among those under 18. Significant differences were observed in age group, clinical stage, and survival status between ICU and non-ICU groups (p < 0.05). The overall mortality rate was 5.7% (15/261), with 4.3% (13/30) among the ICU group and 0.9% (2/231) among the non-ICU group.

**Table 1:** Distribution of demographic and clinical data of COVID-19 patients between non-ICU and ICU groups. ^a ^Chi-square test; p < 0.05 is statistically significant.

Characteristics	Non-ICU (n=231)	ICU (n=30)	p-value
Age group			
<18	6 (2.6%)	0 (0.0%)	<0.05^a^
18-35	78 (33.8%)	0 (0.0%)	
36-55	66 (28.6%)	8 (26.7%)	
>55	81 (35.1%)	22 (73.3%)	
Gender			
Male	97 (42.0%)	16 (53.3%)	0.238
Female	134 (58.0%)	14 (46.7%)	
Clinical stage			
Stage 1	11 (100%)	0 (0.0%)	<0.05^a^
Stage 2	48 (100%)	0 (0.0%)	
Stage 3	85 (100%)	0 (0.0%)	
Stage 4	87 (85.3%)	15 (14.7%)	
Stage 5	0 (0%)	15 (100%)	
Survival status			
Alive	229 (99.1%)	17 (56.7%)	<0.05^a^
Death	2 (0.9%)	13 (43.3%)	

Significant differences in mean values for certain hematological parameters were observed between the ICU and non-ICU groups (p < 0.05). Specifically, the ICU group showed significantly lower mean values for lymphocyte and eosinophil counts, as well as the LMR. Conversely, the ICU group exhibited significantly higher mean values for WBC, neutrophil, and IG counts, as well as the NLR and PLR. The detailed findings are presented in Table 2.

**Table 2:** Comparison of routine and extended hematological parameters of COVID-19 patients between non-ICU and ICU groups. ^a^ Independent t-test; p < 0.05 is statistically significant. ICU: intensive care unit; WBC: white blood cells; Hb: hemoglobin; IG: immature granulocyte; HFLC: highly fluorescent lymphocyte cells

Hematological parameters	Non-ICU (n=231)	ICU (n=30)	
	Mean (SD)	Mean (SD)	p-value
WBC (x 10^3^/µL)	5.92 ± 2.31	9 ± 5.1	0.003^a^
Hb (g/dL)	13.12 ± 2.02	13.14 ± 2.4	0.952
Platelet (x 10^3^/µL)	232.41 ± 87.22	221 ± 80.18	0.496
Neutrophil (x 10^3^/µL)	3.47 ± 1.89	7.41 ± 5.01	<0.001^a^
Lymphocyte (x 10^3^/µL)	1.81 ± 0.68	1.08 ± 0.33	<0.001^a^
Monocyte (x 10^3^/µL)	0.57 ± 0.28	0.5 ± 0.25	0.155
Eosinophil (x 10^3^/µL)	0.05 ± 0.07	0	<0.001^a^
Basophil (x 10^3^/µL)	0.01 ± 0.01	0 ± 0.01	0.074
IG (%)	0.34 ± 0.21	0.65 ± 0.39	<0.001^a^
HFLC (%)	0.77 ± 0.65	0.68 ± 0.6	0.461
Neutrophil lymphocyte ratio	2.12 ± 1.19	7.69 ± 5.78	<0.001^a^
Lymphocyte monocyte ratio	3.73 ± 1.84	2.67 ± 1.32	0.002^a^
Platelet lymphocyte ratio	140.3 ± 60.32	223.9 ± 106.52	<0.001^a^

The model predicting ICU admission and survival status in COVID-19 patients is depicted through simple and multiple logistic regression analysis on hematological parameters (Table 3). Eosinophil and basophil counts were omitted from the analysis due to data skewness and failure to meet statistical criteria. Simple logistic regression analysis identified several potential independent factors associated with ICU admission, including WBC, neutrophil, lymphocyte, IG, NLR, LMR, and PLR. Similarly, factors associated with survival among COVID-19 patients were identified as WBC, neutrophil, IG, and NLR. Variables exhibiting statistical significance (p < 0.05) underwent multiple logistic regression to select variables for inclusion in the predictive model, aiming to identify the association of combined hematological parameters with both ICU admission and survival status following 30 days of COVID-19 infection.

**Table 3:** Simple and multiple logistic regression analysis for potential factors associated with ICU admission and survival among COVID-19 patients. ^a ^p < 0.05 is statistically significant. ICU: intensive care unit; CI: Confidence Interval; WBC: white blood cells, Hb: hemoglobin, HFLC: highly fluorescent lymphocyte cells, NLR: neutrophil-to-lymphocyte ratio, LMR: lymphocyte-to-monocyte ratio, PLR: platelet-to-lymphocyte ratio, IG: immature granulocyte

Analysis	Category	ICU admission				Patients’ survival			
Simple logistic regression	Parameters	p-value	Odd ratio	95% CI		p-value	Odd ratio	95% CI	
				Lower	Upper			Lower	Upper
	WBC	<0.001^a^	1.302	1.165	1.454	0.008^a^	0.838	0.735	0.954
	Hb	0.951	1.006	0.835	1.211	0.224	1.158	0.914	1.467
	Platelet	0.494	0.998	0.994	1.003	0.438	1.003	0.996	1.009
	Neutrophil	<0.001^a^	1.457	1.273	1.667	0.001^a^	0.810	0.713	0.920
	Lymphocyte	<0.001^a^	0.060	0.019	0.184	0.115	2.070	0.837	5.118
	Monocyte	0.157	0.325	0.069	1.542	0.089	8.320	0.722	95.809
	IG	<0.001^a^	37.858	9.082	157.818	<0.001^a^	0.061	0.013	0.282
	HFLC	0.460	0.787	0.417	1.486	0.658	1.216	0.512	2.891
	NLR	<0.001^a^	2.159	1.592	2.928	0.001^a^	0.835	0.749	0.930
	LMR	0.003^a^	0.634	0.468	0.858	0.649	0.938	0.714	1.234
	PLR	<0.001^a^	1.013	1.008	1.018	0.370	0.997	0.991	1.003
Multiple logistic regression	WBC	<0.001^a^	0.084	0.03	0.232	0.014^a^	36.677	2.086	644.889
	Neutrophil	<0.001^a^	23.959	7.296	78.675	0.011^a^	0.023	0.001	0.425
	Lymphocyte	-	-	-	-	0.028^a^	0.018	0.001	0.644
	IG	-	-	-	-	0.032^a^	0.12	0.017	0.831

Multiple logistic regression analysis emerged WBC and neutrophil counts as significant predictors for ICU admission, with a 1 x 10^3/L increase in neutrophil count corresponding to a 23.96 times higher odds of ICU admission, even after adjusting for WBC. In addition, a similar analysis revealed that WBCs, neutrophils, lymphocytes, and IG counts were important predictors for patient survival in COVID-19 infection. WBC was the most significant predictor, with an odds ratio of 36.677 and 95% CI [2.086, 644.889], indicating that patients with WBC count exceeding 1 x 10^3/µL had 36.68 times higher odds of survival, after adjusting for neutrophil, lymphocyte, and IG levels.

To assess model fit and discriminatory ability, the area under the ROC curve was calculated. Statistically significant associations were observed between WBCs and neutrophil counts and ICU admission, and between WBC, neutrophil, lymphocyte, and IG counts and patient survival among COVID-19-infected patients (Tables 4). Other hematological parameters were not statistically significant and were excluded from the final model.

**Table 4:** Diagnostic accuracy of prognostic factor in prediction of ICU admission and survival among COVID-19 patients (n=261). ICU: intensive care unit; CI: Confidence Interval; WBC: white blood cells; IG: immature granulocyte; AUC: area under the curve

Category	Prognostic factors/final model	AUC (95% CI)	Lower CI	Upper CI	Sensitivity (%)	Specificity (%)	p-value
ICU admission	Combination of WBC, and neutrophil counts	0.92	0.867	0.972	83.3	84.0	<0.001
Patients’ survival	Combination of WBC, neutrophil, lymphocyte, and IG counts	0.781	0.642	0.92	73.2	73.3	<0.001

**Figure 1:**
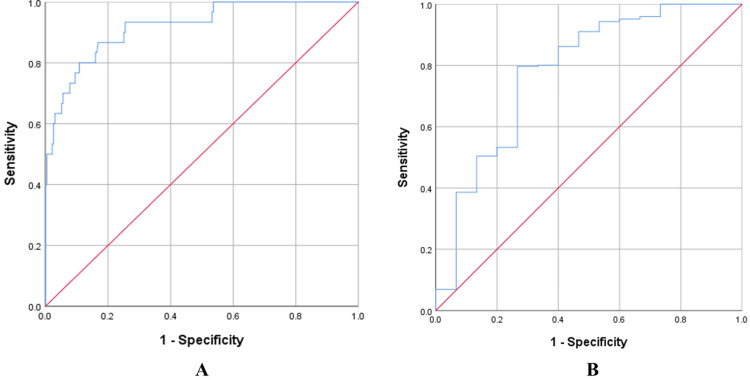
(A) The ROC curve of a combination of WBC and neutrophil counts for predicting ICU admission; (B) The ROC curve of a combination of WBC, neutrophil, lymphocyte, and IG counts for predicting the survival of COVID-19 patients. ROC: receiver operating characteristic curve; WBC: white blood cells; IG: immature granulocyte

The ROC curve analysis showed an AUC of 0.920 (95% CI: 0.867-0.972; p < 0.001) for ICU admission and 0.781 (95% CI: 0.642-0.920; p < 0.001) for survival among COVID-19 patients (Figures 1A-1B, respectively). These curves accurately discriminated 92% of COVID-19 cases with a chance for ICU admission and 78.1% of cases with a chance for survival.

## Discussion

The majority of COVID-19 recruited patients in the present study aged 55 years or older (39.46%), slightly higher than a previous study (38%) [9] and the reported mortality rate was 5.7% deviates slightly from earlier studies (2.5-5.0%) [10,11]. However, Minnai et al. reported a significantly higher overall mortality rate of 23.1% among hospitalized COVID-19 patients [11]. The difference could be attributed to age, as Minnai et al.'s study included a significant number of old age patients, particularly those over 85 years old compared to our data.

Our study revealed a significantly higher mortality rate of 43.3% among ICU patients, with the majority aged over 55, representing a slight increase compared to the Czech Republic study reporting 37.0% mortality [12]. In Iran, ICU mortality stood at a prominent 68%, with the highest mortality observed in patients over 62 years old [13]. Moreover, a meta-analysis of 59 articles emphasized a strong correlation between ICU admission and patients aged over 70 [14] underscoring age as a crucial determinant of COVID-19 progression and outcomes [15].

The severity of COVID-19 infection is determined by both clinical observations and laboratory data [14]. In Malaysia, COVID-19-confirmed patients are categorized into five groups according to guidelines from the Ministry of Health, Malaysia [16]. Our findings indicated that most ICU-admitted patients were classified as clinical stages 4 and 5, necessitating ventilator support. In contrast, patients in the non-ICU group were predominantly classified as clinical stages 1 to 3. Among ICU-admitted patients, 26.7% (8 out of 30) in the age group of 36 to 55 years old and 73.3% (2 out of 30) in the age group over 55 years old required ventilation support. In line with our findings, the previous study has shown that a higher percentage of COVID-19 patients in clinical stages 4 and 5 require mechanical ventilation, particularly among males and elderly individuals aged over 70 [14].

Upon comparing the laboratory results of COVID-19 patients, significant differences were found in WBCs, neutrophils, lymphocytes, eosinophils, IG counts, NLR, LMR, and PLR between non-ICU and ICU groups. Specifically, WBCs, neutrophil, IG counts, NLR, and PLR were significantly higher in the ICU group, while the other parameters were lower. This aligns with the findings of a study that included 117 COVID-19 patients, which also showed statistically significant mean differences between regular and severely critically ill groups [17]. Moreover, our study's findings regarding NLR, LMR, and PLR were consistent with Asghar et al. [18].

NLR stands out as a significant hematological parameter in COVID-19 patients, characterized by an elevated absolute neutrophil count alongside a low absolute lymphocyte count. A meta-analysis by Chan and Rout identified the NLR as an independent prognostic marker for COVID-19. Their study found that NLR levels were five times higher in severe cases compared to non-severe cases, effectively distinguishing between the two categories [19]. Other studies have shown that severe COVID-19 cases exhibit a mean NLR of 9.9, higher than mild cases (2.08), indicating a direct association between NLR and disease severity [18,20]. In our study, ICU cases showed a higher NLR of 7.69 ± 5.78 compared to 2.12 ± 1.19 in non-ICU cases, consistent with previous findings. This observation can be explained by the central role of neutrophils in the innate immune response. Initially, neutrophilia occurs as neutrophils form neutrophil extracellular traps, activating the immune system and releasing reactive oxygen species that induce cellular damage and virus release. Neutrophils also trigger the production of cytokines and complements, amplifying the inflammatory response and leading to a cytokine storm. Consequently, COVID-19 is associated with systemic inflammation and elevated interleukin 6 levels, resulting in reduced lymphocyte counts [20]. Previous studies investigating lymphopenia's association with COVID-19 severity have reported lower absolute lymphocyte counts in severe cases [21]. Both factors contribute to an elevated NLR. Therefore, based on our findings, a higher NLR can serve as a valuable marker for predicting inflammation severity in COVID-19 patients [22].

This study observed a significantly lower eosinophil count in the ICU group compared to the non-ICU group, consistent with findings in 78.8% of COVID-19-positive patients particularly among critically ill patients at higher risk of mortality [23]. Additionally, an increasing eosinophil count has been described as potentially predicting a favorable clinical outcome in COVID-19 patients [24]. However, Asghar et al. found no significant difference in eosinophil count between ICU and non-ICU patients. It's worth noting that they measured eosinophil count in percentage value rather than absolute count [18].

A study examined the peripheral blood film (PBF) of 78 COVID-19 patients, with the majority (81.5%) in the ICU found that 85.2% of patients had IG in their PBF, representing promyelocytes, myelocytes, and metamyelocytes. Similarly, Martens et al. explored the hematological features of COVID-19 patients with cytokine storm syndrome (CSS), finding a higher percentage of IG count [25]. These results align with our findings, showing a 0.31% higher IG count in the ICU group. This increase may be due to infection-induced bone marrow stress, particularly from CSS seen in COVID-19 patients [26].

For the LMR, we found a significantly lower mean difference between ICU and non-ICU groups, which is consistent with a previous study involving 24 severe COVID-19 patients [10]. Furthermore, Gong et al. demonstrated that reduced LMR is associated with inflammatory conditions [27]. Collectively, these observations suggest the significant role of lymphocytes in the regulatory pathway of the immune system, with a lower lymphocyte count potentially linked to inflammation resulting from the apoptotic process [28].

Like the previous study, our PLR exhibited significantly higher mean differences in the ICU group [10]. Interestingly, high PLR showed an association with disease severity in COVID-19 patients [29]. Despite the high PLR observed in severe COVID-19 patients, PLR was a less significant predictor of disease severity when compared with NLR [30]. In assessing COVID-19 infection, both low LMR and high PLR offer valuable additional information. Our analysis revealed a noteworthy association between low LMR and high PLR among patients progressing to a critically ill condition requiring ICU admission.

Previous studies have noted higher ICU admission rates among patients with elevated WBCs, neutrophil, lymphocyte, and IG counts, but their predictive performance has been inadequate for accurately identifying patients at risk of severe outcomes. Interestingly, our model achieved high accuracy, with an AUC of 0.92 for predicting ICU admission risk and 0.78 for survival status. These findings also align with previous studies, indicating a correlation with COVID-19 severity. Our ROC curve analysis underscores the potential of this final model as a robust set of hematological predictors for severe COVID-19 cases, particularly considering the widespread availability of hematological analyzers in many hospitals.

In line with the advanced technology nowadays, two well-established clinical calculators for assessing COVID-19 severity are available online, such as the COVID-19 Estimator (https://covidest.web.app/calculator.html) and the COVID-19 Hospitalization Risk Calculator (https://riskcalc.org/COVID19Hospitalization/). These tools use various parameters to predict hospitalization and mortality risks, including age, sex, vaccination status, SARS-CoV-2 variant, body mass index (BMI), hypertension, hemoglobin A1C, chronic obstructive pulmonary disease (COPD), immune deficiency, and kidney function (glomerular filtration rate (GFR)). Healthcare providers can use the COVID-19 Estimator to quickly evaluate patients' risk levels, aiding in triage and prioritizing those who might need intensive care. Additionally, the COVID-19 Hospitalization Risk Calculator assesses the likelihood of hospitalization and severe outcomes based on demographics, underlying health conditions, and vital signs. This tool may assist in identifying high-risk patients who may benefit from early intervention and closer monitoring upon hospital admission.

Interestingly, our study highlights the significant predictive value of hematological markers such as WBC and neutrophil counts for ICU admission, and WBC, neutrophil, lymphocyte, and IG counts for survival status among COVID-19 patients. Integrating these hematological markers with established clinical calculators may enhance their predictive accuracy and serve as a promising strategy for improving the prediction of ICU admissions in COVID-19 patients. This approach can enhance clinical decision-making, ensuring patients receive appropriate care based on a comprehensive risk assessment. By combining these methods, healthcare providers can better manage patient care and allocate resources more effectively. Therefore, future research should focus on validating and calibrating these calculators to incorporate hematological markers, ensuring they reflect the latest clinical evidence and provide reliable predictions across various healthcare settings.

This study has notable limitations. Firstly, data were collected from a single center, potentially restricting the generalizability of our findings compared to multicenter studies. Additionally, as a hospital-based study, asymptomatic COVID-19 patients were excluded, limiting direct comparisons with community-level data. As a result, further validation through larger-scale studies is necessary.

## Conclusions

This study underscores the critical role of hematological parameters in predicting ICU admission and survival status among COVID-19 patients. Elevated WBC and neutrophil counts emerged as the most significant predictors for ICU admission and overall survival, respectively. These findings highlight the importance of early and precise identification of patients at higher risk for severe disease progression, which can facilitate timely interventions and better allocation of healthcare resources. Our results provide a valuable framework for incorporating hematological markers into clinical protocols to enhance the management of severe respiratory infections, including COVID-19.

Therefore, this study recommends hospitals and healthcare facilities consider integrating routine hematological screenings for COVID-19 patients upon admission. This practice can help identify high-risk patients who may require more intensive monitoring and early intervention. Medical staff should be trained to interpret hematological parameters effectively. Additionally, healthcare systems should ensure adequate resources and equipment for comprehensive blood testing and analysis.
